# Proteome-wide survey of phosphorylation patterns affected by nuclear DNA polymorphisms in *Arabidopsis thaliana*

**DOI:** 10.1186/1471-2164-11-411

**Published:** 2010-07-01

**Authors:** Diego Mauricio Riaño-Pachón, Sabrina Kleessen, Jost Neigenfind, Pawel Durek, Elke Weber, Wolfgang R Engelsberger, Dirk Walther, Joachim Selbig, Waltraud X Schulze, Birgit Kersten

**Affiliations:** 1 Max Planck Institute of Molecular Plant Physiology, Am Mühlenberg 1, 14476 Potsdam-Golm, Germany; 2 Institute for Biochemistry and Biology, University of Potsdam, Karl-Liebknecht-Straße 24-25, Haus 20, 14476 Potsdam-Golm, Germany; 3 Johann Heinrich von Thuenen-Institute, Federal Research Institute for Rural Areas, Forestry and Fisheries, Institute for Forest Genetics, Sieker Landstr. 2, 22927 Grosshansdorf, Germany; 4 Institute of Pathology, Universitätsmedizin Charite, Chariteplatz 1, 10117 Berlin, Germany; 5 Department of Computer Science, Humboldt University of Berlin, Rudower Chaussee 25, 12489 Berlin, Germany

## Abstract

**Background:**

Protein phosphorylation is an important post-translational modification influencing many aspects of dynamic cellular behavior. Site-specific phosphorylation of amino acid residues serine, threonine, and tyrosine can have profound effects on protein structure, activity, stability, and interaction with other biomolecules. Phosphorylation sites can be affected in diverse ways in members of any species, one such way is through single nucleotide polymorphisms (SNPs). The availability of large numbers of experimentally identified phosphorylation sites, and of natural variation datasets in *Arabidopsis thaliana *prompted us to analyze the effect of non-synonymous SNPs (nsSNPs) onto phosphorylation sites.

**Results:**

From the analyses of 7,178 experimentally identified phosphorylation sites we found that: (i) Proteins with multiple phosphorylation sites occur more often than expected by chance. (ii) Phosphorylation hotspots show a preference to be located outside conserved domains. (iii) nsSNPs affected experimental phosphorylation sites as much as the corresponding non-phosphorylated amino acid residues. (iv) Losses of experimental phosphorylation sites by nsSNPs were identified in 86 *A. thaliana *proteins, among them receptor proteins were overrepresented.

These results were confirmed by similar analyses of predicted phosphorylation sites in *A. thaliana*. In addition, predicted threonine phosphorylation sites showed a significant enrichment of nsSNPs towards asparagines and a significant depletion of the synonymous substitution. Proteins in which predicted phosphorylation sites were affected by nsSNPs (loss and gain), were determined to be mainly receptor proteins, stress response proteins and proteins involved in nucleotide and protein binding. Proteins involved in metabolism, catalytic activity and biosynthesis were less affected.

**Conclusions:**

We analyzed more than 7,100 experimentally identified phosphorylation sites in almost 4,300 protein-coding loci *in silico*, thus constituting the largest phosphoproteomics dataset for *A. thaliana *available to date. Our findings suggest a relatively high variability in the presence or absence of phosphorylation sites between different natural accessions in receptor and other proteins involved in signal transduction. Elucidating the effect of phosphorylation sites affected by nsSNPs on adaptive responses represents an exciting research goal for the future.

## Background

Protein phosphorylation is one of the most important and best characterized post-translational modification (PTM) among the hundreds so far described [1-3]. Reversible site-specific phosphorylation of serine (S), threonine (T), or tyrosine (Y) by concerted actions of kinases and phosphatases plays a central role in virtually all cellular processes [4], especially in cell signaling of prokaryotes and eukaryotes, including plants [5,6]. Phosphorylation of a protein can occur at multiple sites and can be catalyzed by different kinases, often in crosstalk with other types of PTM [1,7,8]. Phosphorylation/dephosphorylation of specific amino acids in proteins can have profound effects on protein structure, activity, stability, subcellular localization and interaction with other biomolecules [4], and it can create binding sites for specific modular domains [9]. Interestingly, in the flowering plant *Arabidopsis thaliana*, the percentage of genes predicted to encode protein kinases (3% of the predicted proteome) is about twice as high as in mammals [10,11]. Protein phosphorylation events have been found to be connected with the plant's response to diverse intrinsic and extrinsic factors, such as light, invasion of pathogens, hormones, temperature stress, and nutrient starvation [6,12,13].

Recent progress in mass spectrometry (MS)-based technologies and phosphopeptide enrichment methods have allowed to map *in vivo *phosphorylation sites for a wide variety of organisms in a high throughput manner [12-14]. This progress has prompted the creation of dedicated web-resources in the plant field, such as PhosPhAt [15] and P3DB [16]. The availability of experimentally verified *A. thaliana *phosphorylation sites now enables *in silico *analyses of different phosphorylation site patterns on a proteome-wide scale. Recently, the conservation of protein phosphorylation sites within selected gene families could be shown in different plant species [7,17]. In non-plant species, where the databases of phosphorylation sites are much more comprehensive than in *A. thaliana*, the *in silico *analysis of phosphoproteomic data has already produced interesting insights into evolutionary features of protein phosphorylation [18-21].

A loss of a single phosphorylation site by a non-synonymous (ns) single nucleotide polymorphism (SNP) that mutates the amino acids S, T, or Y at a phosphorylation site into any other amino acid can have profound effects on the molecular properties of the corresponding protein. In particular, the disruption of phosphorylation sites by such non-synonymous mutations can be associated with human diseases such as cancer. For example, the phosphorylation of T286 in wildtype cyclin D1 by the kinase GSK3B initiates its nuclear export and subsequent degradation in the cytoplasm [22]. The authors suggested that the loss of this phosphorylation site by a somatic mutation is involved in causing nuclear accumulation of cyclin D1 in esophageal cancer and a generally increased oncogenic potential.

In *A. thaliana*, genomic DNA polymorphisms have been studied extensively during the last few years, initially using gene expression microarrays in order to identify single-feature polymorphisms (SFPs) [23]. Especially SNPs, in several inbreed accessions can be applied to study the effects of these polymorphisms on other genome-wide features, such as phosphorylation sites.

Given the importance of protein phosphorylation, we were interested to study how phosphorylation sites and their patterns are affected by natural variation, namely nsSNPs in *A. thaliana*. In this study we analyzed the distribution of all phosphorylation sites taken from the recent version of PhosPhAt (version 3.0) [15] in the proteome of *A. thaliana *and we related their position to nsSNPs thus identifying losses of phosphorylation sites. For that purpose, we made use of *A. thaliana *SNPs identified recently by applying re-sequencing arrays [24,25] and by re-sequencing with ultra-deep sequencing technologies such as Illumina/Solexa [26]. Our aim was to analyze how the *A. thaliana *phosphorylation sites and their patterns are influenced by these nsSNPs.

Because the current dataset of experimental phosphorylation sites in *A. thaliana *is far away from covering the entire proteome, the results obtained from the experimental phosphorylation site dataset were contrasted with results produced by similar analyses of predicted phosphorylation sites in *A. thaliana *to attain more global hypotheses on protein phosphorylation patterns and the influence of nsSNPs on them.

## Results

### Description of the phosphorylation site sets used in this study

Experimental phosphorylation sites from different phosphoproteomic studies in *A. thaliana *[27-39] were taken from PhosPhAt (version 3.0) [15] comprising a total of 7,178 unambiguously identified phosphopeptides mapping to 4,252 protein-coding loci. The proportional frequency of phosphorylated S, T, Y residues (pS, pT, pY) in this experimental set was similar to distributions reported previously, with 70.7% of pS, 20.7% pT, and 8.6% pY [34].

We were able to confirm that the majority of phosphorylation sites (71%) occur outside conserved protein domains, as noted previously [34]. Specifically, pS and pT occurred within domains in only 22.4% and 36.8% of the cases. However, phosphorylated tyrosines were located inside protein domains in 49.8% of the cases. Similar behavior was observed for the set of predicted phosphosites (data not shown).

The set of predicted phosphorylation sites used in this study was also taken from PhosPhAt (version 3.0) [15]. It comprised 75,296 high-confidence phosphopeptides (score ≥1; see Methods), identified in 21,711 protein-coding loci. The relative frequency of phosphorylated S, T, Y residues in this set of predicted phosphorylation sites was 37.0% pS, 42.5% pT and 20.5% pY.

Based on our sets of experimentally identified as well as high-confidence predicted phosphorylation sites (score ≥1), we looked for under- and overrepresented plant GO Slim terms among different sets of phosphoproteins: i) all proteins containing at least one pS; ii) all proteins containing at least one pT; iii) all proteins containing at least one pY; iv) all proteins containing at least one pS or at least one pT (p[ST]); v) all proteins containing at least one phosphorylated residue (p[STY]) (Additional file 1 for experimental phosphorylation sites; Additional file 2 for predicted phosphorylation sites). A comparison of the GO annotation of the p[STY]-protein sets based on experimental and predicted sites is shown in the Figure included in Additional file 3. In both, experimental and predicted datasets, the terms "catalytic activity", "kinase activity", "transferase activity", and "protein modification process" were overrepresented among proteins containing p[STY] sites. Also, several stress-related terms were found overrepresented in both. In experimental as well as predicted p[STY]-protein sets, proteins with function in "translation", "RNA binding", "biosynthetic process" were determined as underrepresented. However, the plant GO Slim terms "transcription factor activity" and "transcription regulator activity" were found overrepresented among the predicted p[STY]-protein set, while underrepresented in the experimental set.

For both, experimental and predicted phosphorylation sites, we then computed the agreement of GO Slim terms between protein sets representing different sites or combination of sites. We evaluated the significance (corrected p-value, FDR ≤5E-2) of the correlation between the following pairs of datasets: pS-pT, pS-pY, pS-p[ST], pS-p[STY], pT-pY, pT-p[ST], pT-p[STY], pY-p[ST], pY-p[STY]. For each dataset in the pair we registered whether a given GO Slim term was present or absent, and then compared the obtained binary series using the Pearson's correlation coefficient. None of the compared profiles were correlated in the experimental dataset, but in the dataset of predicted phosphoproteins we found a moderate correlation of overrepresented plant GO Slim term profiles between pS-p[ST] and pY-pT, with correlation coefficients of 0.39 and 0.38, respectively.

### Distribution of phosphorylation sites across proteins

**Figure 1:**
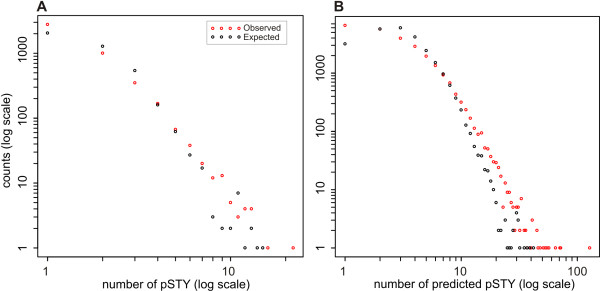
**Frequency distribution of the count of the number of phosphorylation sites per protein**. To compute the expected distribution of phosphorylation sites per protein (black circles) we assumed that that every possible STY-site becomes phosphorylated based on a constant probability *p*, which is independent on the number of STY sites per protein and was obtained by dividing the total number of pSTY-sites by the total number of STY positions across all proteins in the data set, *p *= total number_pSTY/total number_STY. With *p *available, the expected number of phosphorylation sites per protein was computed as E(pSTY)_x _= *p *x Number of_SYT in protein X. The observed distribution of phosphorylation sites per proteins appears as red circles (A: Experimental phosphorylation sites; B: high-confidence predicted phosphorylation sites).

Most of the *A. thaliana *phosphoproteins contained only a few experimental phosphorylation sites, whereas a few proteins were phosphorylation hubs with a large number of phosphorylation sites. Noticeably, there is a long tail of the distribution of the number of phosphosites per protein, i.e., the proportion of proteins with a large number of phosphorylation sites is higher than expected by chance alone. This is especially evident for the predicted phosphosites. Additionally, proteins with a single phosphorylation site appear more often than expected, for both the experimental sites and the predicted sites, highlighting the physiological importance of phosphorylation (Figure 1; Additional file 4). Proteins with a large number of phosphorylation sites are of special interest as potential sites of integration (hubs) in regulatory pathways.

Table 1 lists the 31 proteins with nine or more experimentally determined phosphorylation sites. One third of these proteins are involved in metabolic processes associated with nucleic acids (Gene Ontology term GO: 6139; from the biological process ontology), especially with RNA splicing (nine out of the ten proteins). Three out of those nine proteins were also identified to include hotspots of experimental phosphorylation sites for a window size of 10 amino acids (see below and Table 2), namely the following proteins: AT5G52040.1, AT5G64200.1, AT3G55460.1. The protein with the most phosphorylation sites was ATRSP41, an arginine/serine-rich splicing factor 41 (AT5G52040.1) which had 22 experimentally verified phosphorylation sites. In a human study, the serine/arginine repetitive matrix protein 2 (SRRM2) with even 142 pS sites holds the top of the list of human proteins with the highest number of pS sites [18]. With progressing identification of *A. thaliana *phosphorylation sites in future studies and under various environmental conditions, we expect that also some *A. thaliana *proteins might be detected to bear even more phosphorylation sites than the 22 sites found in ATRSP41.

**Table 1:** Proteins with 9 or more experimentally identified phosphorylation sites in *A. thaliana*. Proteins with phosphorylation hotspots appear in italics. ^*a*^Proteins annotated with the GO term "nucleobase, nucleoside, nucleotide and nucleic acid metabolic process" (GO: 6139).

AGI	Number of phosphorylation sites	Number of p(S);p(T);p(Y)	Protein length	TAIR7 function
*AT5G52040.1*^*a*^	22	(19;1;2)	357	ATRSP41 (Arabidopsis thaliana arginine/serine-rich splicing factor 41); RNA binding

AT2G29210.1^*a*^	16	(13;1;2)	879	Splicing factor PWI domain-containing protein

AT2G37340.1^*a*^	13	(13;0;0)	291	RSZ33 (Arginine/serine-rich Zinc knuckle-containing protein 33); nucleic acid binding/nucleotide binding/zinc ion binding

*AT5G64200.1*^*a*^	13	(13;0;0)	304	ATSC35 ("Arabidopsis thaliana arginine/serine-rich splicing factor 35, 35 kDa protein"); RNA binding

AT2G43680.1	13	(11;2;0)	669	IQD14; calmodulin binding

*AT3G55460.1*^*a*^	13	(11;0;2)	263	SCL30 (SC35-like splicing factor 30); RNA binding

*AT1G35580.1*	12	(10;2;0)	552	CINV1 (CYTOSOLIC INVERTASE 1); beta-fructofuranosidase

AT3G25500.1	12	(12;0;0)	1052	AFH1 (FORMIN HOMOLOGY 1); actin binding

AT3G63400.1^*a*^	12	(11;1;0)	571	Peptidyl-prolyl cis-trans isomerase cyclophilin-type family protein

AT5G47690.1	12	(10;2;0)	1606	Binding

AT3G23900.1	11	(11;0;0)	988	RNA recognition motif (RRM)-containing protein

AT2G18960.1	11	(5;4;2)	950	AHA1 (PLASMA MEMBRANE PROTON ATPASE); ATPase

AT2G20960.1	11	(4;7;0)	749	pEARLI4

*AT3G53500.1*^*a*^	10	(9;0;1)	244	RSZ32; nucleic acid binding

*AT4G33240.1*	10	(8;2;0)	1758	1-phosphatidylinositol-4-phosphate 5-kinase/zinc ion binding

*AT5G01400.1*^*a*^	10	(7;3;0)	1468	ESP4 (ENHANCED SILENCING PHENOTYPE 4); binding

AT1G31870.1	10	(10;0;0)	562	Similar to splicing factor PWI domain-containing protein [Arabidopsis thaliana] (TAIR:AT2G29210.1)

AT5G47430.1	10	(9;0;1)	893	Unknown function

AT4G32420.1	9	(8;0;1)	838	Peptidyl-prolyl cis-trans isomerase cyclophilin-type family protein

AT5G10470.1	9	(9;0;0)	1274	Kinesin motor protein-related

AT4G02510.1	9	(8;1;0)	1504	TOC159 (translocon outer membrane complex 159)

*AT4G31160.1*	9	(7;2;0)	1847	Transducin family protein/WD-40 repeat family protein

AT3G26935.1	9	(6;2;1)	444	Zinc finger (DHHC type) family protein

AT5G47910.1	9	(9;0;0)	922	RBOHD (RESPIRATORY BURST OXIDASE PROTEIN D)

AT5G61150.1	9	(9;0;0)	626	VIP4 (VERNALIZATION INDEPENDENCE 4)

AT5G43310.1	9	(5;4;0)	1238	COP1-interacting protein-related

AT5G40450.1	9	(8;1;0)	2890	Unknown function

*AT3G13570.1*^*a*^	9	(8;1;0)	263	SCL30a (SC35-like splicing factor 30a); RNA binding

AT3G61860.1^*a*^	9	(8;0;1)	265	ATRSP31 (ARGININE/SERINE-RICH SPLICING FACTOR 31); RNA binding

AT1G48920.1	9	(8;1;0)	558	Nucleolin, putative

AT1G19870.1	9	(9;0;0)	795	IQD32 (IQ-domain 32); calmodulin binding

**Table 2:** *A. thaliana *proteins with potential phosphorylation hotspots consisting of experimental phosphorylation sites in a window of 10 amino acids.

AGI	Number of significant windows	Number of phospho-sites in window of 10 amino acids (window start)	TAIR7 function
AT4G07523.1	4	5(6), 6(4), 4(7), 5(1)	Unknown function

AT4G33240.1	4	4(1538), 4(1545), 4(1557), 5(1543)	1-phosphatidylinositol-4-phosphate 5-kinase/zinc ion binding

AT1G53165.1	3	4(442), 4(439), 5(441)	Kinase

AT2G46170.1	3	4(22), 5(28), 4(29)	Reticulon family protein (RTNLB5)

AT3G04650.1	3	4(4), 5(3), 4(1)	Oxidoreductase

AT1G35580.1	2	4(66), 4(44)	CINV1 (CYTOSOLIC INVERTASE 1); beta-fructofuranosidase

AT3G18180.1	2	4(67), 4(69)	Unknown function

AT1G08680.1	1	4(191)	ZIGA4 (ARF GAP-LIKE ZINC FINGER-CONTAINING PROTEIN ZIGA4); DNA binding

AT1G29220.1	1	4(81)	Transcriptional regulator family protein

AT1G55310.1	1	4(5)	SR33 (SC35-like splicing factor 33); RNA binding

AT1G70130.1	1	4(281)	Lectin protein kinase, putative

AT1G73200.1	1	4(313)	Unknown function

AT2G26730.1	1	4(632)	Leucine-rich repeat transmembrane protein kinase, putative

AT2G35880.1	1	4(109)	Unknown function

AT2G41705.1	1	4(61)	Camphor resistance CrcB family protein

AT2G46495.1	1	4(402)	Zinc finger (C3HC4-type RING finger) family protein

AT3G27960.1	1	4(574)	Kinesin light chain-related

AT3G29310.1	1	4(325)	Calmodulin-binding protein-related

AT3G29390.1	1	4(512)	RIK (RS2-INTERACTING KH PROTEIN)

AT3G48530.1	1	4(14)	CBS domain-containing protein

AT3G55460.1	1	4(176)	SCL30 (SC35-like splicing factor 30); RNA binding

AT3G58940.1	1	4(113)	F-box family protein

AT4G14605.1	1	4(290)	Mitochondrial transcription termination factor-related/mTERF-related

AT4G31580.1	1	4(170)	SRZ-22 (serine/arginine-rich 22)

AT4G32250.1	1	4(22)	Protein kinase family protein

AT5G02240.1	1	4(235)	Catalytic/coenzyme binding

AT5G14890.1	1	4(60)	NHL repeat-containing protein

AT5G52040.1	1	4(342)	ATRSP41 (Arabidopsis thaliana arginine/serine-rich splicing factor 41); RNA binding

AT5G64200.1	1	4(274)	ATSC35 ("Arabidopsis thaliana arginine/serine-rich splicing factor 35, 35 kDa protein"); RNA binding

### Phosphorylation hotspots within proteins

For proteins with many experimentally identified phosphorylation sites, we analyzed the location of those sites within the protein, to identify potential hotspots of phosphorylation.

Based on the experimental sites, we determined several hotspot-containing proteins using window sizes of 5, 10, 15, and 20 amino acids (Additional file 5). We then applied the phosphorylation hotspot analysis to the predicted phosphorylation site dataset to get a more global view on potential phosphorylation hotspots in the *A. thaliana *proteome. For predicted sites as well, we were able to determine several phosphorylation hotspots in proteins for window sizes of 10, 20, 30 and 40 amino acid residues (Additional file 6 and 7).

**Figure 2:**
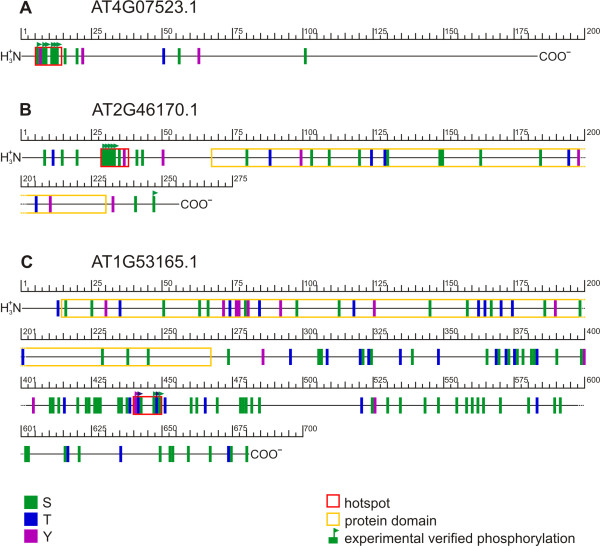
**Phosphorylation hotspots in *A. thaliana *proteins**. Hotspots in three *A. thaliana *proteins which were identified based on the analysis of experimental phosphorylation sites with a window size of 10 amino acids (Additional file 5). Hotspots are indicated by red boxes. A: AT4G07523.1 represents a protein of unknown function (TAIR7). B: AT2G46170.1 is annotated as reticulon family protein (TAIR7). C: AT1G53165.1 was found to be a protein kinase (TAIR7). Amino acid residues S, T and Y are marked by green, blue and purple rectangles. Rectangles with a flag represent experimentally verified phosphorylation sites. Pfam identified protein domains are highlighted by yellow boxes.

Using experimental phosphorylation sites, and for a window size of ten amino acids, 43 potential phosphorylation hotspots in 29 different proteins were identified (Table 2). Figure 2 shows three hotspots with the highest number of experimental phosphorylation sites (six or five pS sites) in a window of 10 amino acids. These hotspots were identified in a protein of unknown function (AT4G07523.1), in a reticulon family protein (AT2G46170.1), and in a protein kinase (AT1G53165.1) based on TAIR7 annotation.

In general, phosphorylation hotspots were preferentially located outside conserved protein domains (p-value: 6.88E-4 for experimental phosphoprotein sets, for window size 10; p-value < 1.0E-7 for predicted phosphoprotein sets for window sizes 20, 30, and 40).

To identify over- or underrepresented biological functions among the proteins containing at least one phosphorylation hotspot that consists of experimental phosphorylation sites, these proteins were tested against a reference set which contained all proteins with at least one experimental phosphorylation site. GO enrichment analyses revealed that the GO Slim term "nucleoplasm" (GO: 5654, from the cellular component ontology) was significantly overrepresented (p-value: 1.52E-3 for window size 10). This was confirmed by the proteins with hotspots consisting of predicted phosphorylation sites (p-value: 4.60E-4 for window size 20). Interestingly, among the proteins with predicted phosphorylation hotspots, "catalytic activity" (GO: 3824) was significantly underrepresented (p-value: 2.40E-3 for window size 40).

### Effect of SNPs on proteins

The mapping of SNPs onto coding sequences allowed us to evaluate their effects on the coded amino acids, and thus the expressed proteins. There were 12,285,899 amino acid residues in the non-redundant set of the protein models represented in the reference genome. In total, 314,705 amino acids and 594 stop codons were affected by SNPs (2.56% of all amino acid residues), either in a synonymous (160,634; 1.31%) or non-synonymous (155,311; 1.26%) way.

**Figure 3:**
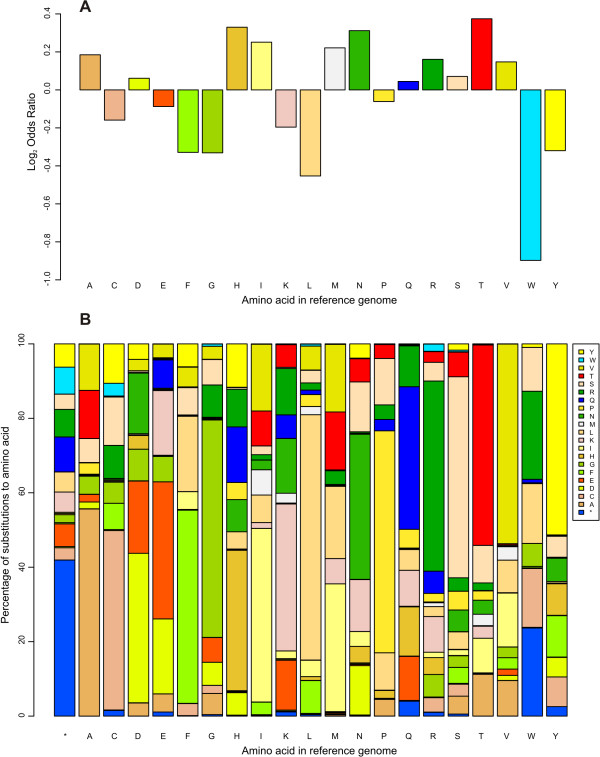
**Amino acids in the reference genome affected by SNPs**. A. Log_2 _odds ratio relating the number of non-synonymous substitutions per amino acid with its abundance in the whole non-redundant proteome. All ratios are significantly different from 1 after Benjamini-Hochberg [63], p-value correction (FDR ≤ 5E-2, see Additional file 8). B. Proportion of amino acids affected by SNPs (synonymous and non-synonymous substitutions).

As expected, there is a moderate positive correlation between the proportion of synonymous substitutions (compared to all substitutions) for each amino acid and the number of codons encoding the respective amino acid (Pearson's correlation coefficient: 0.74; p-value: << 5E-2). In order to account for amino acid abundances throughout the proteome, we performed a 2-way contingency table analysis on the number of non-synonymous substitutions affecting each amino acid and the total number of a given amino acid in the whole non-redundant proteome (Figure 3A, Additional file 8). The strong underrepresentation of L, F, G, W and Y residues being affected by substitutions suggests a stronger global functional or structural constraint on these amino acids. Most SNPs caused synonymous substitutions (Figure 3B).

Interestingly, S and T, two phosphorylatable amino acids, are more likely to be affected by nsSNPs than expected. By contrast, Y residues were 3 to 5 times less frequently affected by SNPs than S or T residues (data not shown).

### Effects of SNPs on phosphorylation sites

**Figure 4:**
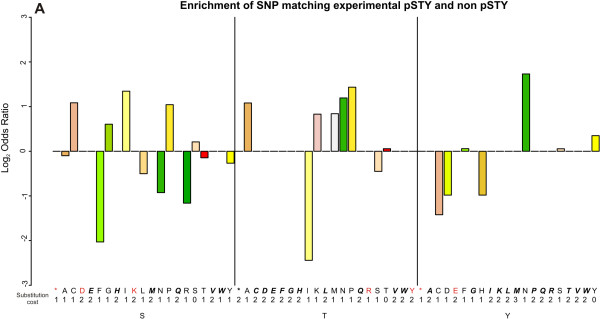
**Effect of SNPs, comparison between experimental phosphorylation sites and non-phosphorylation sites**. We evaluated the enrichment and depletion of each substitution pair from an experimentally identified phosphorylation site to any other amino acid, by using 2-way contingency tables for each pair and evaluating the significance of an odds ratio different from 1 (corrected p-value, FDR ≤ 5E-2) with a Fisher's exact test. All ratios are statistically undistinguishable from 1 (Log Odds = 0). Only experimentally verified phosphoproteins were included in this analysis. Substitution of amino acids in bold were never found, neither in phosphorylation sites nor in non-phosphorylation sites. Substitution amino acids in red were present among non-phosphorylation sites, but absent among phosphorylation sites. The substitution cost is the minimal number of DNA substitutions that is required in order to change an amino acid into another (see Additional file 9).

We tested whether there was any association between substitutions caused by SNPs affecting experimental phosphorylation sites compared to non-phosphorylation [STY] sites, but we did not observe any overall trend (Fisher's exact test p-value: >> 5E-2). We then looked at the effect of each individual amino acid substitution (Figure 4). We found that amino acid substitutions do rarely occur, when requiring two or three nucleotide substitutions (simultaneous changes of 2 or 3 bases in the underlying codon, i.e., substitution cost). The only observed cases were for S to D, S to K, T to Y, and Y to E, although even in these cases, the substitution only occurred in non-phosphorylated S, T or Y residues. We also found that in all cases substitutions were either more frequent in phosphorylated sites than in non-phosphorylated sites or vice versa, never equally affecting both types of sites.

In case of the predicted phosphorylation sites, we found for threonine a significant enrichment of non-synonymous substitutions in phosphorylated compared to non-phosphorylated threonine residues (data not shown). Analyzing the effect of each individual substitution in the predicted dataset, we observed several amino acid substitutions with a substitution cost of two that occurred in phosphorylation sites (S to D, S to K, T to E, T to Q, T to V, Y to E, Y to L and Y to T; Additional file 9 and 10). Regarding the enrichment and depletion of individual substitutions between phosphorylated and non-phosphorylated sites, we found that all substitutions were as likely to occur in predicted phosphorylation sites as in non-phosphorylation sites, except for the substitution T to N, which was more likely to occur in predicted phosphorylation sites than in non-phosphorylation sites, and the synonymous substitution T to T, which was more likely in non-phosphorylation sites. In that respect, it is important to note that asparagine (N) has been shown to mimic a phosphorylated serine, in a mutant of the *A. thaliana *K^+ ^channel AKT2 (AT4G22200.1). Asparagine is an uncharged amino acid, but it is larger than both S and T. Mimicking the phosphorylation site could arise due to steric effects as suggested by Michard *et al*. [40]. Thus, a substitution T to N might generate a constitutive phosphorylation site.

#### Losses of experimental phosphorylation sites

Among the experimental phosphorylation sites, we identified 86 sites (in 86 proteins) that were lost in at least one of the *A. thaliana *accessions by an exchange of a phosphorylation target amino acid S, T, or Y to any other amino acid (Additional file 11). For four proteins (AT1G01550.1, AT1G44800.1, AT1G62330.1, AT5G38310.1) the phosphorylated S was substituted to more than one other amino acid in the accessions analyzed. For example a pS (position 341) of the nodulin MtN21 family protein (AT1G44800.1) was exchanged to T in most of the accessions with the only exception of Bur-0, where an N was introduced by the nsSNP.

Interestingly, we found that among these 86 proteins, the GO Slim categories "receptor activity" was significantly overrepresented, but there were no underrepresented categories. Using a reference set comprising all *A. thaliana *proteins with at least one phosphorylation site, no over- or underrepresented categories could be identified.

Remarkably, some losses of experimentally verified phosphorylation sites are located in phosphorylation hotspots. This concerns the following *A. thaliana *proteins: two proteins of unknown function (AT2G35880.1; pS at position 118 and AT3G18180.1; pS at position 67); a calmodulin-binding protein-related (AT3G29310.1; pS at position 327), and the protein ESP4 (ENHANCED SILENCING PHENOTYPE 4); binding (AT5G01400.1; pS at position 1364), based on TAIR7 annotation.

#### Gains and losses of predicted phosphorylation sites

Using the dataset of predicted high-confidence phosphorylation sites for a more global analysis, we found 1,114 proteins in which predicted phosphorylation sites could potentially be lost in at least one of the *A. thaliana *accessions studied (Additional file 12). In 1,103 cases, the SNP caused an amino acid substitution to an amino acid that cannot be phosphorylated, while in the remaining 11 proteins, the score of the phosphorylation prediction changed from a high-confidence positive score (score ≥1) to a value smaller than or equal to -1, regardless of the type of putative phosphorylated amino acid at the given position in the neighborhood of the affected phosphorylation site. A prediction value of score ≤ -1 is taken as high-confidence prediction of the amino acid not being phosphorylated.

In contrast, we observed that 1,148 proteins gained a predicted phosphorylation site (Additional file 12). The majority of gained phosphorylation sites (1,136) emerged by the change of a non-phosphorylatable amino acid to an S, T, or Y residue and a resulting prediction score ≥1 of the newly generated putative phosphorylation site. In the remaining 12 cases, the gain of the phosphorylation site was based on the score alone, the prediction score increased from a negative score (score ≤ -1) to a score higher than or equal to 1 due to nsSNPs that resulted in amino acid exchanges in the neighborhood to S, T and Y residues thus creating a new phosphorylation site target motif.

We found that the GO categories "receptor activity", "binding" and "signal transducer activity" were significantly overrepresented in all three datasets regardless of the reference set used (see Methods; Additional file 13). Additionally, in proteins which gained a phosphorylation site, and in all proteins with a gain or a loss, the category "response to stress" was overrepresented, regardless of the reference set used (Additional file 13). In the dataset including gain and loss of phosphorylation sites, the categories "transporter activity", "catalytic activity", "metabolic process", "biosynthetic process" and "cell" were underrepresented using the set of predicted phosphoproteins as reference set (Additional file 12). The figure in Additional file 14 presents all over- and underrepresented GO Slim categories in the protein set with losses and gains of predicted phosphorylation sites.

## Discussion

With regard to the isolation of phosphorylated proteins from complex samples as well as their mass spectrometric and computational analysis the progress that has been made in *A. thaliana *is quite impressive. However, the connection of protein phosphorylation with genetic variation in natural occurring ecotypes at a proteome-wide level in plant species has not been addressed so far. In this study we made use of experimental and predicted phosphorylation sites available in PhosPhAt [15] and mapped the data onto the *A. thaliana *genome annotation. This is currently the largest phosphorylation site dataset in *A. thaliana *comprising 7,178 experimentally verified unique phosphorylation sites assigned to 4,252 protein-coding loci. The combined data set represents phosphorylation sites identified from different tissues/cell types representing varied developmental stages and responses.

Since the assigned proteins cover only one-sixth of the predicted *A. thaliana *protein-coding loci, we extended our study by also including predicted phosphorylation sites to achieve better proteome coverage in our analyses, attaining a coverage of 80% of all protein-coding loci in *A. thaliana*.

The relative frequency of 70.7% pS, 20.7% pT, and 8.6% pY in our experimental set was similar to distributions reported previously in *A. thaliana *([34]; 85.0% pS, 10.7% pT, and 4.3% pY) and in humans [41,42]. In the predicted dataset, the fraction of tyrosine residues was much higher (25.0%) compared to the experimental set (8.6%). This discrepancy may result from a bias in the training set of experimental phosphopeptides that has been used for the phosphorylation site predictor or from a lack of accuracy of the predictor. In addition, we cannot exclude that the experimentally identified phosphorylation sites may in general be biased by experimental restrictions such as specific phosphopeptide enrichment methods [43] or by a focus on specific stress conditions or subcellular compartments. Thus, this distribution may still shift by including new phosphorylation sites from future studies. However, since in general experimental and predicted data show similar distribution among and within the proteins, the inclusion of predicted data in the scope of this study is justified.

Multisite phosphorylation in proteins has been discussed as points of integration of different signal transduction pathways [1,44]. However, a major difficulty in studying multisite phosphorylation from a system's perspective has been the uncertainty whether simultaneous or successive phosphorylation occurred at the different sites on the same protein and whether the multiple sites were phosphorylated by the same or different protein kinases. Dependencies among phosphorylation sites in a single protein can be intricate [45], and the position and the number of phosphorylated residues can affect the biological outcome [46].

In the experimental dataset, we observed that the identified number of phosphorylation sites per protein was largely in agreement with the expected values, except for large values of phosphorylation sites and a single phosphorylation site. This observation was more noticeable for the predicted dataset. This suggests that there are at least three discrete regions in the distribution: i) a region of overrepresentation of single phosphorylation sites, ii) a region where the number of phosphorylation sites is proportional to the total number of S, T and Y; i.e., the more sites can be phosphorylated, the more will and proportionally, and, iii) a region where phosphorylation sites appear more often than expected given the total number of S, T, Y. Similar behavior was previously suggested to result from a rich-gets-richer process for the accumulation of phosphorylation sites [18], however the low coverage of the experimental dataset in this study does not allow us to arrive at such a conclusion. Nevertheless, the data in Figure 1, strongly suggests that once a single phosphorylation event happened in a protein, further phosphorylations will accumulate depending only on the abundance of phosphorylatable residues, until reaching a threshold whereafter further phosphorylations will happen even more rapidly than dictated by the abundance of phosphorylatable residues (longer tail).

Our finding that the phosphorylation hotspots occur preferentially outside conserved domains suggests that, indeed, they may serve as sites of signal integration as they are outside of regions such as catalytic domains or protein-protein interaction domains. Multisite phosphorylation occurring outside structured regions was shown for single nuclear proteins in several studies. This is the case for the human protein Ets-1. Multiple Ca^2+^-dependent phosphorylation sites in an unstructured flexible region of this transcription factor act additively to produce graded DNA binding affinity [47]. Our result that nucleus-related GO terms are overrepresented in hotspot-containing proteins is in line with studies that indicate a central involvement of many nuclear proteins as integration hubs for phosphorylation-dependent signaling [48].

In an MS-based phosphoproteomics study in *A. thaliana*, it was suggested that the mRNA splicing machinery is a major target of protein phosphorylation [29]. Our results support this hypothesis. One third (10/31) of the proteins with nine or more experimentally determined phosphorylation sites are involved in metabolic processes associated with nucleic acids, especially with "RNA splicing" (Table 1).

Disease resistance genes, S-locus proteins and receptors had previously been shown to display a high variability between different wild varieties of *A. thaliana *[23]. In combination with our findings this indicates that receptors belong to the more variable proteins in *A. thaliana *accessions, and gains or losses of phosphorylation sites in rapidly evolving and variable regions of receptors could facilitate the evolution of kinase-signaling circuits [49]. The importance of specific phosphorylation sites in *A. thaliana *receptor proteins for receptor dimer formation and activation of signaling events was concluded from experiments on the BRI1/BAK1 receptors [50]. Similarly, in different human receptor proteins the importance of their site-specific phosphorylations could be demonstrated, for example in receptor tyrosine kinases of the ErbB family [51].

The potential contribution of the identified losses and gains of the phosphorylation sites by nsSNPs to adaptive responses of the various natural accessions will be an interesting field to be analyzed in future association studies.

## Conclusions

By mapping nsSNPs onto phosphorylation sites, we identified losses and gains of phosphorylation sites, which can be important in adaptive responses of the natural accessions in their different environments. Especially receptor proteins were affected by losses of experimental phosphorylation sites. Based on the observed gains and losses of predicted phosphorylation sites it can be expected that beyond receptor proteins also other proteins involved in signaling and stress response are affected by changes, whereas proteins involved in metabolism, catalytic activity and biosynthesis are less affected. These findings suggest a relatively high variability of signal transduction-related proteins and receptors and more conserved regulation in metabolism. Since receptors and signaling processes are primarily involved in recognition and response to environmental cues, the overrepresentation of phosphorylation sites (gain or loss) in these functional classes indeed supports the view of nsSNPs as evolutionary means of adaptation.

## Methods

### Genome annotation and identification of protein domains

The *A. thaliana *Columbia-0 genome was sequenced in the year 2000 [52]. In this study, we used the genome annotation provided by The Arabidopsis Information Resource, release 7.0 (TAIR7) [53]. Protein domains were identified using the Pfam v23 library of Hidden Markov Models [54]. 2,902 Pfam HMMs have significant hits in 19,904 protein-coding loci (23,760 protein models) in TAIR7.

### Phosphoproteomics datasets

#### Experimental phosphorylation sites

Experimental phosphorylation sites of different phosphoproteomic studies in *A. thaliana *[27-36] were taken from PhosPhAt (version 3.0) [15]. The PhosPhAt database aims to collect manually curated phosphorylation motifs from *A. thaliana*. While PhosPhAt provides both, phosphorylation sites with uncertain and exactly defined positions within the respective protein sequence, only the latter were included in this study. The dataset comprised 7,178 unambiguously identified phosphopeptides identified in 4,252 protein-coding loci annotated in TAIR7 (with the following counts per residue type: 5,078 S; 1,482 T; 618 Y).

#### Predicted phosphorylation sites

The predictions of phosphorylation of S, T, and Y were extracted from PhosPhAt. PhosPhAt uses a Support Vector Machine (SVM) in order to reliably predict phosphorylation sites in *A. thaliana*. The SVM has been trained on experimentally verified phosphorylation sites from *A. thaliana *with the goal of specifically capturing the properties of plant phosphorylation sites and was shown to predict plant phosphorylation sites with a considerably better performance than other available predictors usually trained on non-plant species [15].

### SNP datasets

Three datasets of SNPs, with polymorphisms detected in the *A. thaliana *accessions listed in Additional file 15 were used in this study. The first large scale SNP study was published in 2005. We refer to it here as the Nordborg2005 dataset. 20,667 non-redundant SNPs were identified in this study in 96 accessions [25]. Clark *et al*. used re-sequencing arrays to identify 1,126,176 non-redundant SNPs in 20 accessions; 637,522 non-redundant SNPs belong to the high-confidence set and were kept for further analysis (Clark2007 in the following) [24,55]. Ossowski *et al*. used ultra-deep sequencing and identified 860,154 non-redundant SNPs in three accessions (Ossowski2008 in the following) [26,56]. SNP positions determined in the datasets Nordborg2005, Clark2007 and Ossowski2008 were combined into a non-redundant dataset that comprised 1,247,284 SNPs (Table 3). 25% of these SNP positions can be mapped onto coding sequences, and around 50% of those lead to an amino acid substitution in at least one of the *A. thaliana *accessions studied (Table 3). The merged SNP dataset used in this study, including SNPs mapping onto *A. thaliana *cDNAs (TAIR7), is publicly available and downloadable via The GABI Primary Database (GabiPD; http://www.gabipd.org/) [57]. Moreover, SNPs can be interactively visualized in the cDNA sequences in the *A. thaliana *Gene GreenCards in GabiPD, where accession-related SNP configuration is provided.

**Table 3:** Overview of the number of SNPs per dataset.

Dataset	Number of non-redundant SNPs in this study	Number of non-redundant SNPs mapping onto cDNAs	Number of non-redundant SNPs mapping onto CDS	Number of non-redundant SNPs causing at least one non-synonymous substitution	Number of non-redundant SNPs always causing synonymous substitutions
Nordborg2005	20,667	9,251	8,023	4,047	3,975

Clark2007	637,522	263,718	227,497	109,709	117,788

Ossowski2008	860,154	220,984	174,559	84,400	90,159

TOTAL	1,247,284	382,770	315,039	156,034	159,004

### Mapping SNP positions onto the TAIR7 genome release and annotation

The datasets Nordborg2005 and Clark2007 were originally mapped onto previous versions of the *A. thaliana *genome sequence, thus the first step in the present study was to bring all three datasets to refer to the same reference coordinate system, i.e., TAIR7. We took the 30 basepairs (bp) of right and left flanking sequences of each SNP position, together with the SNP base, from the reported *A. thaliana *genome versions. Using MEGABLAST (W = 39, D = 3) [58,59] we mapped them onto the TAIR7 genome sequence, allowing only 100% identical matches. None of the polymorphic positions were changed between the different releases of the *A. thaliana *genome. Subsequently, using the genome annotation provided by TAIR7, we determined which of these polymorphic positions occurred inside processed mRNAs and protein coding sequences. The latter is necessary to determine if the SNPs caused a synonymous or non-synonymous substitution, and allowed us to determine, which phosphorylation site positions were affected by SNPs.

### Evaluating enrichment of gene ontology terms in sets of proteins

The gene ontology (GO) provides a set of controlled and structured vocabularies, i.e., ontologies, in three domains of molecular biology: cellular component, biological process and molecular function [60]. A large proportion of *A. thaliana *genes in TAIR7 have been annotated with at least one GO term. In this study, we used a subset of these GO terms, known as the plant GO Slim, which provides a high level view of the above mentioned ontologies. Over- and underrepresentation analysis of plant GO Slim terms was carried out using the plugin BiNGO v2.3 [61] for the software package Cytoscape [62]. Statistically significant categories, either over- or underrepresented, were selected according to their corrected *p*-value (False Discovery Rate (FDR) ≤ 5E-2), using a hypergeometric test.

### Effect of SNPs on phosphorylation sites

#### Differences between the effects of SNPs on phosphorylation sites and non-phosphorylation sites

We evaluated the differences between SNPs affecting phosphorylation- and non-phosphorylation sites by comparison of their distributions. For each natural accession from *A. thaliana*, we mapped all SNPs separately on the respective protein sequences.

Subsequently experimental sites were mapped onto the protein sequences, in which at least one SNP was found. In a third step, the distribution of SNPs mapping to phosphorylation sites and non-phosphorylation sites was evaluated for protein sequences with at least one SNP mapping onto an experimental phosphorylation site. For each of the potentially phosphorylated amino acid S, T and Y (phosphorylation sites and non-phosphorylation sites), we counted the number of synonymous and non-synonymous substitutions to all 20 amino acids and to stop-codons. We also counted the number of S, T, and Y phosphorylation sites and the total number of S, T, and Y in all proteins with at least one SNP mapping onto an experimental phosphorylation site. Based on this information, we created 2 × 2 contingency tables for each substitution pair and evaluated the significance (corrected p-value, FDR ≤ 5E-2) with a Fisher's exact test for each contingency table. Finally, we represented the enrichment and depletion of substitutions between phosphorylation sites and non-phosphorylation sites as the odds ratio between their probabilities.

The same procedure was applied to the sets of predicted phosphorylation sites.

#### Losses of experimental phosphorylation sites

The number of losses of experimental phosphorylation sites was computed by determining SNPs mapping to experimental phosphorylation sites and resulting in an exchange to a non-phosphorylatable amino acid.

#### Gains and losses of predicted phosphorylation sites

SNPs of predicted phosphorylation sites resulting in exchange of S, T or Y to a non-phosphorylatable amino acid were defined as "loss", and SNPs leading to an exchange of a non-phosphorylatable amino acid with a S, T, Y predicted to be phosphorylated with high confidence (decision value ≥1) were defined as "gain". Additionally, we also determined gains and losses of phosphorylation sites by SNPs considering the sequential context between -6 and +6 amino acids around the central residue (S, T, or Y). This sequential context can be affected by SNPs thus changing the probability of phosphorylation of the central S, T or Y. We used only positions with highly confident decision values ≥1 for phosphorylation sites and ≤ -1 for non-phosphorylation sites in the TAIR7 protein sequence considering the accession specific sequence. Thus, there are two types of gains/losses of predicted phosphorylation sites, (i) based on score, where the score of a phosphorylatable amino acid changes from ≥1 to ≤ -1 (loss) or vice versa (gain) due to an amino acid substitution within the phosphorylation site recognition motif, (ii) based on a change from one amino acid residue with high-confidence phosphorylation prediction score into a non-phosphorylatable amino acid (loss) or the creation of a peptide with a confidence prediction score greater than 1 (gain).

Three different datasets were used for an analysis of over- and underrepresentation of plant GO Slim terms among the proteins affected by gain or loss of phosphorylation sites: the first dataset contained only proteins that lost a predicted site; the second dataset consisted of proteins, which gained a predicted site; and the third dataset included proteins with both, gain and loss of predicted phosphorylation sites. These three datasets were compared against two reference sets, (i) a reference set that comprised all proteins containing a predicted phosphorylation site (score ≥ 1) and (ii) a reference set that comprised all *A. thaliana *proteins.

### Identification of phosphorylation hotspots

Hotspots were computed based on two datasets: (i) experimentally verified phosphorylation sites, (ii) predicted phosphorylation sites. A hotspot was defined as a window of a given length, which (i) contains a significantly increased number of phosphorylation sites (for experimental sites) or (ii) has a significantly increased windows score (for predicted sites) compared to an empiric background distribution. Analyses were run using the following window sizes: (i) windows of 5, 10, 15, 20 amino acids length for experimental sites and (ii) windows of 10, 20, 30, 40 for predicted sites.

To generate background proteomes, proteins were sampled according to the length distribution and amino acid composition of all proteins in the *A. thaliana *proteome. Resulting proteins were randomly phosphorylated at S, T, and Y residues with probabilities as identified after mapping all experimental phosphorylation sites onto *A. thaliana *protein sequences in case of experimental sites. For predicted sites, a score was randomly assigned to the S, T, and Y residues by sampling a score from the corresponding distribution of prediction scores in the *A. thaliana *proteome. In this way, 10,000 background proteomes of size of the experimental set were generated for the experimental sites, 1,000 background proteomes of size of the *A. thaliana *proteome were generated for the predicted sites.

To generate empiric background distributions, each background proteome was analyzed by scanning each protein with a window of fixed size. Window scores were computed by using (i) the number of contained phosphorylated amino acid residues for experimental sites, or (ii) the sum of all scores in a window for predicted sites. For a given window size the empiric background distribution is formed by the distribution of window scores. Windows containing the same S, T, and Y residues of a protein were counted only once.

The proteome of *A. thaliana *with mapped experimental or predicted sites was scanned and window scores were determined accordingly. For predicted sites, only windows containing at least the expected number of S, T, and, Y sites based on the determined amino acid frequency in the *A. thaliana *proteome were taken into account for further analysis (in contrast to the background distribution).

The null-hypothesis of statistical testing of hotspots states: the score of a candidate window is derived from the described background distribution. The alternative hypothesis states: If the p-value of the score from a candidate window is less than the significance level (5E-2), then it is assumed that the window results from an unknown hotspot distribution. The right tail of background distribution is considered for testing. P-values were determined and Bonferroni corrected for multiple testing. Windows which had scores with corrected p-value less than 5E-2, were saved for further analysis.

### Overlap of hotspots with protein domains

We tested the hypothesis whether hotspots are uniformly distributed across proteins, independently of protein domains. For the statistical test, (i) the number of hotspots overlapping with a protein domain was computed for the real data, (ii) the same number of hotspots of a given size was sampled onto the proteins, (iii) the hotspots overlapping with domains were counted and saved. This procedure was repeated for 10E7 times and the number of overlaps of the real data was compared to the empirically generated background distribution. The null-hypothesis was tested analogous as described in the previous section.

## Authors' contributions

DMRP and SK carried out statistical analysis and improved and extended the database schema to store and query SNP and phosphorylation site data. JN carried out the phosphorylation site hotspot analysis. PD predicted phosphorylation sites and participated in the analysis of the data. EW performed preliminary analyses and setup of the database to query SNP and phosphorylation site data. WRE and WXS determined experimental phosphorylation sites. DW, JS, WXS and BK designed and coordinated the project. BK led the team. All authors participated in writing the manuscript, read and approved the final manuscript.

## Supplementary Material

Additional file 1 **This file contains seven worksheets with the list of over- and underrepresented plant GO Slim terms in the set of experimentally identified phosphoproteins, determined by BiNGO analysis**. The worksheets S, T, Y, ST and STY contain the tables witht over- and underrepresented GO Slim categories with the following columns: *GO term*; *p-value*; *corrected p-value*; *x*: number of genes in the query dataset annotated to a certain GO term; *X*: the total number of genes in the query dataset, genes without any annotation are discarded; *n*: number of genes in the reference dataset annotated to a certain GO term; *N*: total number of genes in the reference dataset, genes without any annotation are discarded. The worksheets "underrepresented" and "overrepresented" include a presence-/absence comparison of the significant GO terms in the different datasets. Every pair of binary series was compared using the Pearson's correlation coefficient. Click here for file

Additional file 2 **This file contains seven worksheets with the list of over- and underrepresented plant GO Slim terms in the set of high-confidence predicted phosphorylation sites (score ≥ 1), determined by BiNGO analysis**. The worksheets S, T, Y, ST and STY contain the tables witht over- and underrepresented GO Slim categories with the following columns: *GO term*; *p-value*; *corrected p-value*; *x*: number of genes in the query dataset annotated to a certain GO term; *X*: the total number of genes in the query dataset, genes without any annotation are discarded; *n*: number of genes in the reference dataset annotated to a certain GO term; *N*: total number of genes in the reference dataset, genes without any annotation are discarded. The worksheets "underrepresented" and "overrepresented" include a presence-/absence comparison of the significant GO term in the different datasets. Every pair of binary series was compared using the Pearson's correlation coefficient. Click here for file

Additional file 3 **Figure of under- and overrepresented plant GO Slim terms in the experimental (A) and predicted (B) p[STY] dataset**. The BiNGO corrected p-values are given in logarithmic scale. Underrepresented categories are shown in red as negative values, and overrepresented terms are shown in blue as positive values. In cases where the corrected p-value was 0, the number 100 has been arbitrarily assigned as the value shown in the graphic. Click here for file

Additional file 4 **This file contains two worksheets: Experimental: With the number of S + T + Y and the number of observed experimental phosphorylation sites per protein**. Predicted: With the number of S + T + Y and the number of high-confidence (score ≥ 1) predicted phosphorylation sites per protein. It was used to create Figure 1 in the main text. Click here for file

Additional file 5 **This file contains all phosphorylation hotspots, which result from the hotspot analysis of all experimental phosphorylation sites in *A. thaliana *protein sequences**. The analysis was run for different window sizes (5, 10, 15, 20 amino acids), available as different worksheets. AGI (TAIR7): *A. thaliana *gene identifier code according to TAIR 7.0; Number of phosphorylation sites: number of experimentally verified phosphorylation sites in the significant window; hotspot start (aa): start of the hotspot in the protein sequence in amino acids; winsize: size of the analyzed window in amino acids; p-value: the Bonferroni-corrected p-value of the window derived from the background distribution. The significance level for the entire series of tests (α) was set to 5E-2. Click here for file

Additional file 6 **This file contains all phosphorylation hotspots, which result from the hotspot analysis of all predicted phosphorylation sites in *A. thaliana *protein sequences**. The analysis was run for different window sizes (10, 20, 30, 40 amino acids), available as different worksheets. AGI (TAIR7): *A. thaliana *gene identifier code according to TAIR 7.0; position (aa): start position of significant window in protein sequence in amino acids (aa), winsize: size of the analyzed window in amino acids, #STY: number of S,T,Y in significant window; # sigwin: number of significant windows in the AGI; score sum: sum of all prediction scores (SVM decision values) for phosphorylatable sites in the window; sequence: sequence of the significant window; aa(position):score: scored amino acids with related position and score are shown; function (TAIR7): gene function according TAIR7 annotation; function (MapMan): gene function according MapMan annotation. Click here for file

Additional file 7 **This file contains all phosphorylation runs derived from the hotspot analysis based on prediction for the analyzed window sizes (10, 20, 30, 40 amino acids), available as different worksheets**. Runs were generated by merging the identified hotspots to non-redundant contiguous sequences (runs). A run is defined as the amino acid sequence of a hotspot if there is no adjacent/overlapping other hotspot present. In cases of two or more overlapping/adjacent hotspots, a run is defined as the sequence representing the combination of those hotspots in the corresponding protein. Each run generated from hotspots is given with the corresponding AGI, start and stop position (in amino acids) as well as the run sequence. Click here for file

Additional file 8 **This file contains a single worksheet with the data used to create **Figure 3A. Click here for file

Additional file 9 **Analyses of enrichment and depletion of substitutions between phosphorylation sites and non-phosphorylation sites**. This file contains two worksheets, 'Experimental phosphorylation sites' and 'Predicted phosphorylation sites', with the data used to create Figure 4 and the figure in the Additional file 10. P-values were adjusted following the procedure of Benjamini-Hochberg [63], significant ratios have an FDR ≤ 5E-2. The substitution cost is the minimum number of substitutions at the DNA level that are required to change one amino acid into another. Click here for file

Additional file 10 **Effect of SNPs, comparison between predicted phosphorylation sites and non-phosphorylation sites**. We evaluated the enrichment and depletion of each substitution pair (from a predicted phospho-residue to any amino acid, only predicted phosphoproteins were included in this analysis) by using 2 × 2 contingency tables for each substitution pair and evaluating the significance of an odds ratio different from 1 applying a Fisher's exact test. (P-values were adjusted following the procedure of Benjamini-Hochberg [63], significant ratios have an FDR ≤ 5E-2). A star on top of a bar indicates that the odds ratio is statistically significantly different from 1. Inf: Substitution occurred in phosphorylation sites and was absent in non-phosphorylation sites, which gives an odds ratio of infinity. Substitution amino acids in bold were never found, neither in phosphorylation sites nor in non-phosphorylation sites. Substitution amino acids in red were present in non-phosphorylation sites, but absent in phosphorylation sites. The substitution cost is the minimal number of DNA substitutions that are required in order to change one amino acid into another (see Additional file 9 for the dataset used to create this figure). Click here for file

Additional file 11 **Losses of experimentally identified phosphorylation sites**. This file contains a single worksheet with the following columns: *AGI*: Arabidopsis Gene Identifier; *Protein position*: position in the protein sequence (TAIR7.0) where the affected a.a. is located; *Status*: Phosphorylated a.a.; *Accession(substitution)*: *A. thaliana *natural accession name and substituted a.a.; *TAIR7 function*. Click here for file

Additional file 12 **This file contains two worksheets, "loss" and "gain" with the list of all phosphorylation sites which gained or lost a predicted phosphorylation site**. The table includes the status of the loss or the gain: "1" means score-based gain or loss and "2" means that the gain or loss was based on a change from an amino acid residue with a high-confidence phosphorylation prediction score into a non-phosphorylatable amino acid (loss) or the creation of a peptide with a confidence prediction score greater than 1 (gain). Further details can be found in "Methods". Click here for file

Additional file 13 **Over- and underrepresented plant GO Slim categories in sets of loss and gain of predicted phosphorylation sites**. As reference sets we used either all *A. thaliana *proteins or a set that comprised all proteins containing at least one high-confidence predicted phosphorylation site (score ≥ 1). This file contains twelve worksheets, the first six represent analysis results gained with the reference set comprising all proteins containing at least one predicted phosphorylation site, the other six have as reference set all *A. thaliana *proteins. For each over- and underrepresented GO Slim category the following columns are given: *GO ID*; *p-value*; *corrected p-value*; *x*: number of genes in the query dataset annotated to a certain GO term; *X*: the total number of genes in the query dataset, genes without any annotation are discarded; *n*: number of genes in the reference dataset annotated to a certain GO term; *N*: total number of genes in the reference dataset, genes without any annotation are discarded. Click here for file

Additional file 14 **Overrepresented (A) and underrepresented (B) GO Slim terms in proteins with predicted gain or loss phosphorylation sites (by nsSNP)**. The dataset was compared to a reference set that comprised all proteins containing a high confidentially predicted phosphorylation site (score ≥ 1). Enrichment analysis of plant GO Slim terms was carried out using the plugin BiNGO v2.3 [61] for the software package Cytoscape [62]. The size of a node is proportional to the number of genes annotated to that node. White nodes represent GO Slim terms that are not significantly over-/underrepresented. Coloured nodes go from yellow to dark orange, representing p-values from 5E-2 to 5E-7. (P-values were adjusted following the procedure of Benjamini-Hochberg [63]). Click here for file

Additional file 15 **This file contains the list of *A. thaliana *accessions with SNP data that were used in this study**. Click here for file
