# Supplementary figures and images for: Proteome-wide survey of phosphorylation patterns affected by nuclear DNA polymorphisms in Arabidopsis thaliana
Source: BMC Genomics. 2010 Jul 1;11:411. doi: 10.1186/1471-2164-11-411 (PMC2996939; doi:10.1186/1471-2164-11-411)

A

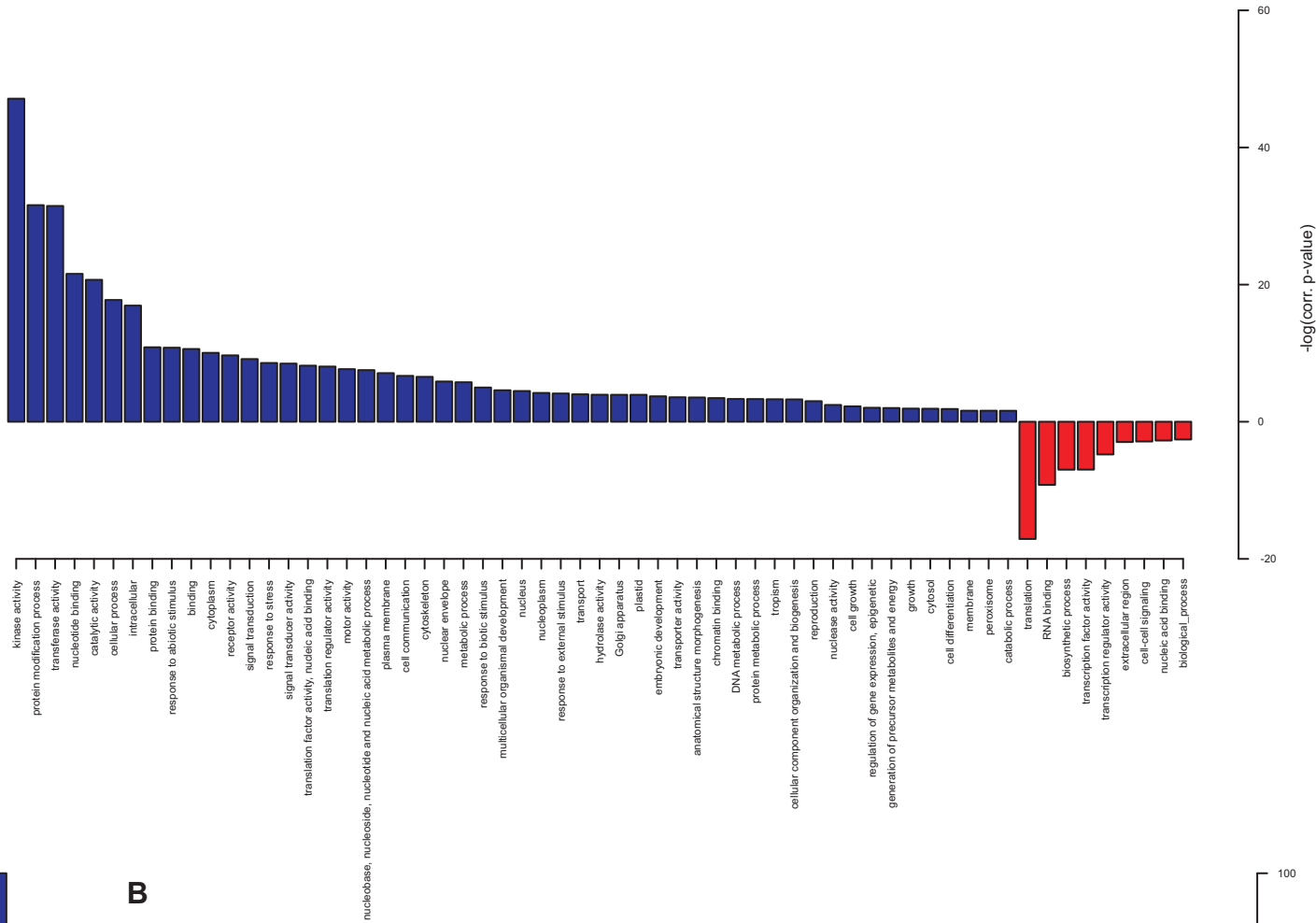

B

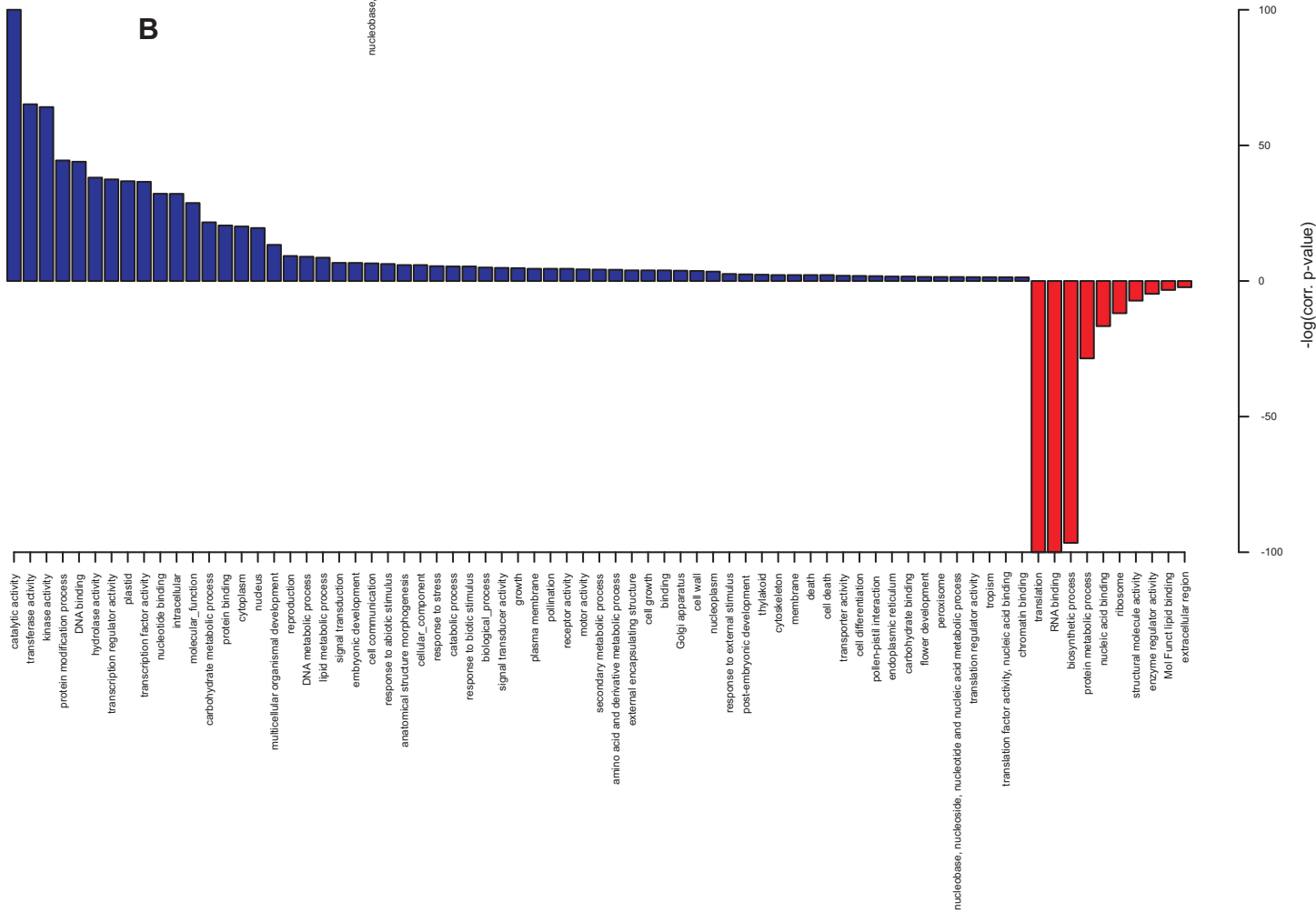

Supplement: Additional file 3 — Figure of under- and overrepresented plant GO Slim terms in the experimental (A) and predicted (B) p[STY] dataset. The BiNGO corrected p-values are given in logarithmic scale. Underrepresented categories are shown in red as negative values, and overrepresented terms are shown in blue as positive values. In cases where the corrected p-value was 0, the number 100 has been arbitrarily assigned as the value shown in the graphic. [file 1471-2164-11-411-S3.PDF]

Enrichment of SNP matching predicted pSTY and non pSTY

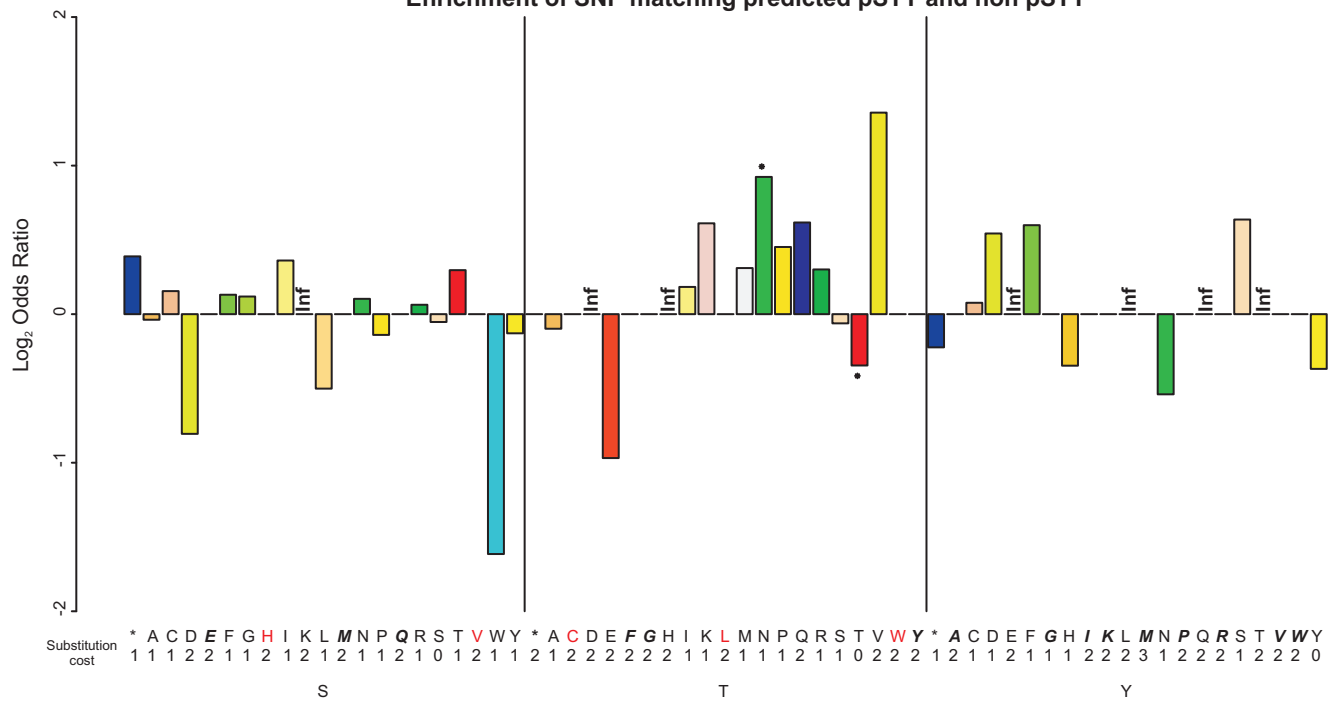

Supplement: Additional file 10 — Effect of SNPs, comparison between predicted phosphorylation sites and non-phosphorylation sites. We evaluated the enrichment and depletion of each substitution pair (from a predicted phospho-residue to any amino acid, only predicted phosphoproteins were included in this analysis) by using 2 × 2 contingency tables for each substitution pair and evaluating the significance of an odds ratio different from 1 applying a Fisher's exact test. (P-values were adjusted following the procedure of Benjamini-Hochberg [63], significant ratios have an FDR ≤ 5E-2). A star on top of a bar indicates that the odds ratio is statistically significantly different from 1. Inf: Substitution occurred in phosphorylation sites and was absent in non-phosphorylation sites, which gives an odds ratio of infinity. Substitution amino acids in bold were never found, neither in phosphorylation sites nor in non-phosphorylation sites. Substitution amino acids in red were present in non-phosphorylation sites, but absent in phosphorylation sites. The substitution cost is the minimal number of DNA substitutions that are required in order to change one amino acid into another (see Additional file 9 for the dataset used to create this figure). [file 1471-2164-11-411-S10.PDF]

A

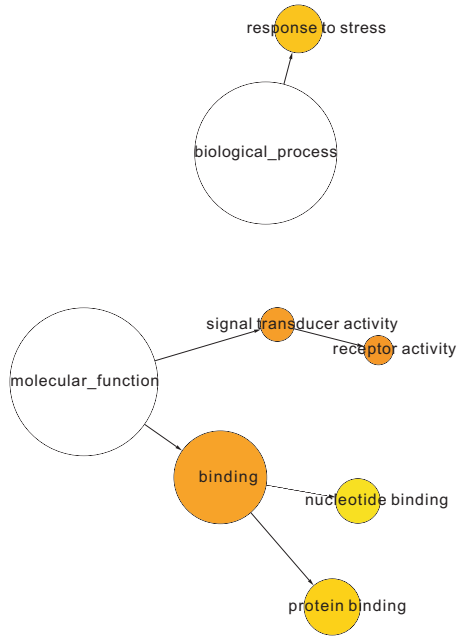

B

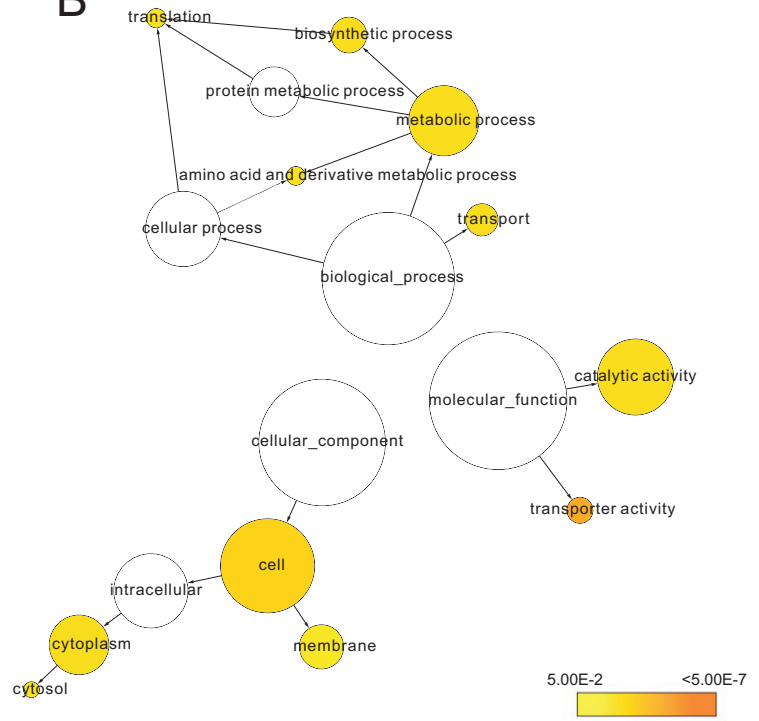

Supplement: Additional file 14 — Overrepresented (A) and underrepresented (B) GO Slim terms in proteins with predicted gain or loss phosphorylation sites (by nsSNP). The dataset was compared to a reference set that comprised all proteins containing a high confidentially predicted phosphorylation site (score ≥ 1). Enrichment analysis of plant GO Slim terms was carried out using the plugin BiNGO v2.3 [61] for the software package Cytoscape [62]. The size of a node is proportional to the number of genes annotated to that node. White nodes represent GO Slim terms that are not significantly over-/underrepresented. Coloured nodes go from yellow to dark orange, representing p-values from 5E-2 to 5E-7. (P-values were adjusted following the procedure of Benjamini-Hochberg [63]). [file 1471-2164-11-411-S14.PDF]
